# Leachability of hexavalent chromium from fly ash-marl mixtures in Sarigiol basin, Western Macedonia, Greece: environmental hazard and potential human health risk

**DOI:** 10.1007/s10653-024-01946-z

**Published:** 2024-04-09

**Authors:** Maria-Nefeli Georgaki, Christina Mytiglaki, Sophia Tsokkou, Nikolaos Kantiranis

**Affiliations:** 1 Environmental Engineering Laboratory, Department of Chemical Engineering, Aristotle University of Thessaloniki, 54124 Thessaloniki, Greece; 2 Department of Mineralogy, Petrology, Economic Geology, School of Geology, Aristotle University of Thessaloniki, 54124 Thessaloniki, Greece; 3 Laboratory of Histology‑Embryology, Department of Medicine, Faculty of Health Sciences, School of Medicine, Aristotle University of Thessaloniki, Thessaloniki, Greece

**Keywords:** Cr (VI), Coal, Fly ash-marl, pH-leaching

## Abstract

Hexavalent chromium (Cr (VI)) is an environmental contaminant brining high concerns due to its higher toxicity and mobility in comparison with trivalent chromium Cr(III). Cr (VI) has been linked with several adverse health effects, including respiratory diseases, lung cancer, and skin irritation. The primary sources of it in the environment are industrial activities.Most of the time, fly ash made of lignite can release Cr(VI) when it comes into contact with water in an aquatic environment. The objective of this study is the investigation of Cr (VI) concentration in leachates of fly ash and marl mixtures and the determination of its solubility under different pH conditions. Samples of fly ash were collected from the Power Plant of Agios Dimitrios. Additionally, samples of marl were collected from the mine of South Field, and both samples were mixed and prepared in in different proportions (% w.t.). The leaching experiments were carried out according to the EN-12457/1-4 (2003) standard under different pH conditions and chemical analysis of the leachates were performed by spectrophotometry with diphenylcarbazide (DPC). The environmental footprint of Cr (VI) in the study area was significant, especially in mixtures containing higher concentrations of fly ash. A critical pH range between 6 to 12 is observed. At acidic pH values, a high release of Cr (VI) was observed, while at the mentioned critical values (pH 10–12), a gradual decrease in its leachability was noticed. The high concentrations of Cr (VI) in the industrial area studied require immediate actions in terms of managing and limiting the potential hazardous impacts on the environment and by extension on the public health by developing appropriate prevention strategies.

**Graphical abstract:**

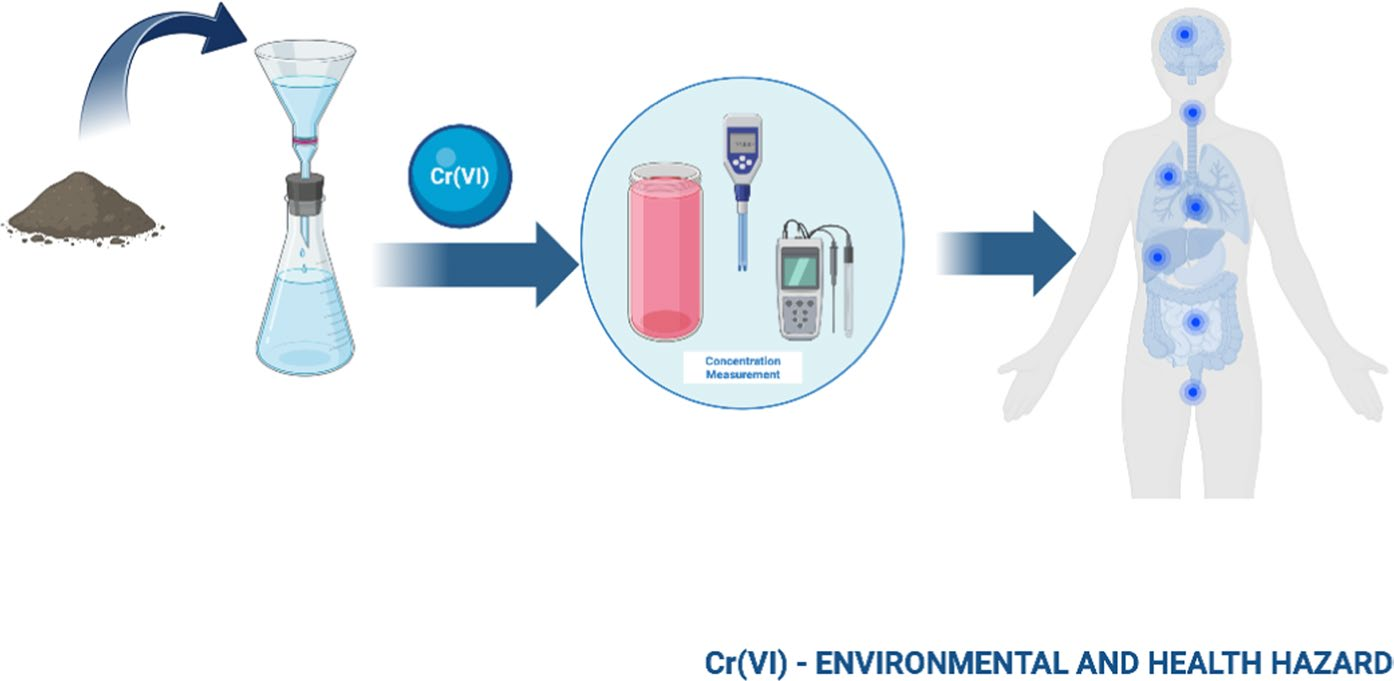

## Introduction

Fly ash, a solid waste from coal combustion contains significant concentrations of potentially dangerous trace elements (Bundschuh et al., 2021; Kamath et al., 2022; Sivapullaiah & Baig, 2010). These trace elements, when exposed to water, are prone to be released in significant quantities into the environment, ending up in aquifers (Deonarine et al., 2015; Eary et al., 1990; Querol et al., 1996). The subsequent release and transport of these elements, either in dissolved or particulate form, can create challenges in both the environment and public health (Amster, 2021; Izquierdo & Querol, 2012). The degree of leaching and solubility of elements in fly ash depends on various factors, including—chemical affinity, solubility of components, combustion conditions, redox conditions, and especially pH conditions prevailing in the area (Komonweeraket et al., 2005; Van der Sloot, 1991).

Chromium (Cr), one of the toxic heavy metals (Kotas & Stasicka, 2000), has been extensively studied in previous environmental studies (Dutta et al., 2009; Fytianos et al., 1998; Warren & Dudas, 1989). Some studies have focused on the high concentration of Cr in soil, surface, or ground water, originated from solid waste, included fly ash. Other studies have examined the combination between Cr and potential health effects. Fly ash leachates frequently contain harmful trace oxyanionic metals like Cr(VI), and due to its disposition on land may be a source of bioavailable chromium, given the global production and disposal of hundreds of millions of tones (Tsiridis et al., 2006; Koukouza et al., 2011). However, despite the extensive research that has been conducted on the degree of mobility and dissolution from fly ash and the assessment of potential environmental impacts on soil and water, it is important to consider which type of Cr (Cr(III) and Cr(VI)) is detected. Although chromium chemistry and leachability have been extensively investigated, few studies have investigated the environmental footprint of Cr(VI), simulating environmental pH conditions, with experimental leaching procedures to determine how this affects fly ash’s overall environmental impact.

Two known forms of Cr can be found in nature—Cr (VI) in the form of chromate salts, or Cr (III) in the form of Cr oxides and hydroxides (Guertin et al., 2005; Kabata-Pendias & Mukherjee, 2007). Their impact is both valuable and harmful towards to human healthdepending on the oxidation state in which Cr is identified (Georgaki et al., 2023). Cr (III) is an essential trace element for human health (Linos et al., 2011; Stern, 2010) and relatively immobile, in comparison with Cr (VI) which is toxic, mutagenic (Costa et al., 1997), and carcinogenic, primarily because of its high mobility and bioavailability (Georgaki et al., 2023). Numerous case studies have demonstrated that long-term exposure to chromium, even at low concentrations, can damage the skin, eyes, respiratory, and immune systems. It can also induce oxidative stress and damage to the DNA, which can accelerate the likelihood of cancers development (Zhang et al., 2014). The World Health Organization (WHO) classifies hexavalent chromium as a highly carcinogenic chemical in group A, linked with stomach cancer, lung cancer, and Hodgkin’s disease (WHO, 2003). Other epidemiological studies have mainly linked hexavalent chromium with issues regarding the liver and kidney function. The type, amount, and length of chromium exposure—which can happen through a variety of routes like food, drink, or inhalation—all impact how the element affects the human body (Georgaki et al., 2023). The increased toxicity and mobility of Cr (VI) in contrast to its trivalent state (Cr (III)) makes it a particularly high-risk environmental pollutant. The occurrence of Cr (VI) in the environment is linked with specific instances influenced by either natural geological processes or human activities. The main factor of the environment’s enrichment with Cr is the geology of each area. Cr concentrations in the earth’s upper crust average 100 mg/kg, with the highest amounts found in basic and ultrabasic rocks (Kukier et al., 2003). In Greece, it is known that the most Cr-enriched rocks are ultramafic and ophiolitic rocks. Cr is mainly found at concentrations of 1000–3000 mg kg^−1^ in ultramafic rocks, together with elements such as Ni, while naturally occurring Cr(VI) is often found in groundwater, which is close to ophiolitic rocks and the products of weathering. conditions resulting from them (Kaprara et al., 2015). Due to the interaction between ultramafic rocks, mainly ophiolitic rocks, and groundwater, the natural environment is burdened with Cr (Kazakis et al., 2017). Numerous studies indicated the high concentration of Cr(VI) as caused by water coming into contact with ultramafic rocks and soils such serpentinite, dunites, and ophiolites (Fantoni et al., 2002; Morrison et al., 2009; Robles-Camacho & Armienta, 2000; Saputro et al., 2014). It is important to mention that the lignite layers become enriched with soluble Cr concentrations, remaining organically bound, as the ultramafic rocks erode (Foscolos et al., 1989; Kaprara et al., 2015).

In addition to the geological origin of Cr, the increase of its concentration, mainly Cr(VI) in soil, surface and groundwater is due to industrial activities. Anthropogenic processes, particularly those associated with mining, coal combustion, processing of Cr-bearing minerals, and disposal of industrial wastes such as fly ash deposition, are primary contributors to the release of Cr(VI) into the environment (Querol et al., 1996; Guertin, 2005). One of the primary Greek lignite mining regions are the Florina-Ptolemais-Kozani basin in northern Greece. The largest power plant in Greece, the Agios Dimitrios Power Plant, is situated in the northern Sarigkiol basin. Due to inadequate management of the fly ash produced, significant quantities of it disperses throughout the surrounding area. It has been reported that Cr(VI) in soils and sediments of the Sarigiol Basin, have been affected by the presence of scattered fly ash, enriched in Cr(VI) as well as the weathering and co-deposition of ultrabasic clastic material with organic and other inorganic materials (Kazakis et al., 2017, 2018). Several studies have been reported regarding the high concentration of Cr identified in the fly ash Characterization of waste—Leachingfrom the Agios Dimitrios Power Plant (Filippidis & Georgakopoulos, 1992; Ibrahim, 2015; Kolovos et al., 2002). The most common type of Cr leached from from fly ash is indicated as Cr(VI) (Iordanidis et al., 2001; Kazakis et al., 2017; Ruppert et al., 1996), while it has been characterized as one of the most toxic trace -metal oxyanions detected in fly ash leachates (Stern, 2010).

Leaching experimental studies provide knowledge concerning the availability and distribution of major and trace elements, including Cr, under specific environmental conditions, estimating potential environmental hazards associated with the disposal or reuse of waste materials (Jegadeesan et al., 2008; Van der Sloot, 1996). Several leaching experiments have been developed to determine the actual harmful effects of the leachability of the potentially toxic components of wastes (Wang et al., 2020). Several established techniques exist, including the EN 12457–2 method protocol, the Gastric Fluid Simulated Test (GJST), the Synthetic Precipitation Leaching Process (SPLP), the pH static Dutch availability test NEN 7341, the Toxicity Characterization Leaching Process (TCLP1), and the Standard Practice for Shaking Extraction of Solid Waste with Water (ASTM D-3987) (NEN7341, 1995; Van der Sloot, 1996; Lu et al., 2009; Jagadeesan et al., 2008). Nevertheless, the intricate chemical processes, during the leaching of Cr, including solubility, complexation, sorption, dissolution/precipitation, and redox reactions, present significant challenges that may compromise the reliability of results. Additionally, the hazardous impact of Cr(VI) may vary depending on parameters such as leaching media, pH conditions, and internal processes in fly ash structures. As such, there may be an inaccuracy in the final outcomes of the research since the results might not precisely reflect the actual environmental conditions (Darakas et al., 2013).

The World Health Organization (WHO, 2020) and the European Union-Drinking Water Directive (98/83/EC) state that the permissible limit for Cr_total_ in drinking water is 50 μg/L. The U.S. Environmental Protection Agency (US EPA, 2010, 2011) has set a similar standard of 100 μg/L for total chromium. There is no specific limit for the hazardous hexavalent form of chromium. Exceptionally, WHO has established a temporary recommendation value of 0.1 μg/L for hexavalent chromium in drinking water, while the European Union has set a maximum discharge limit of 0.5 μg/L for the same element into aquatic environments (Georgaki et al., 2023). Only the Canadian water quality guidelines stipulate a threshold concentration for Cr (VI), of 1 μg/L for Cr (VI) in freshwaters and 1.5 μg/L in seawater (Marine, 1999). It is crucial to emphasize that these recommendations are subjective to changes and should be regularly reviewed and updated.

In this study, particular attention is paid to two industrial by-products—fly ash, and marl. The marl is co-mined and burned with the lignite, so its organic and inorganic components undergo physicochemical changes, and trace elements, such as chromium, are concentrated in fly ash (Kassoli-Fournaraki et al., 1993, Sachanidis et al. 2001). Marl is also used in the mixing and depositing of ash in mine sites (Sachanidis et al. 2001). Investigating the leachability of Cr (VI) in fly ash and marl mixtures is critical given the toxicological and environmental concerns associated with this element. The higher concentration of Cr(VI) in the Sarikiol basin poses a concern to the quality of groundwater resources and, by extension, human health.

The objective of this study was to investigate the leachability of Cr(VI) from samples of fly ash, marl, and their mixtures, under different environmental pH conditions, in Kozani, Greece. The tendency to release this element is studied using various leaching tests, which simulate the relevant environmental conditions. This is the first time that the potential environmental risk of these two materials in mixtures is indicated by evaluating the mobility of Cr(VI) under specific environmental conditions pH as one of the main hazardous elements, in an industrialized area in Greece. Consequently, the results of this study may be used in the future to protect the environment and by extension human health from harmful hexavalent chromium exposure.

## Materials and methods

### Study area (map & geology)

**Fig. 1:**
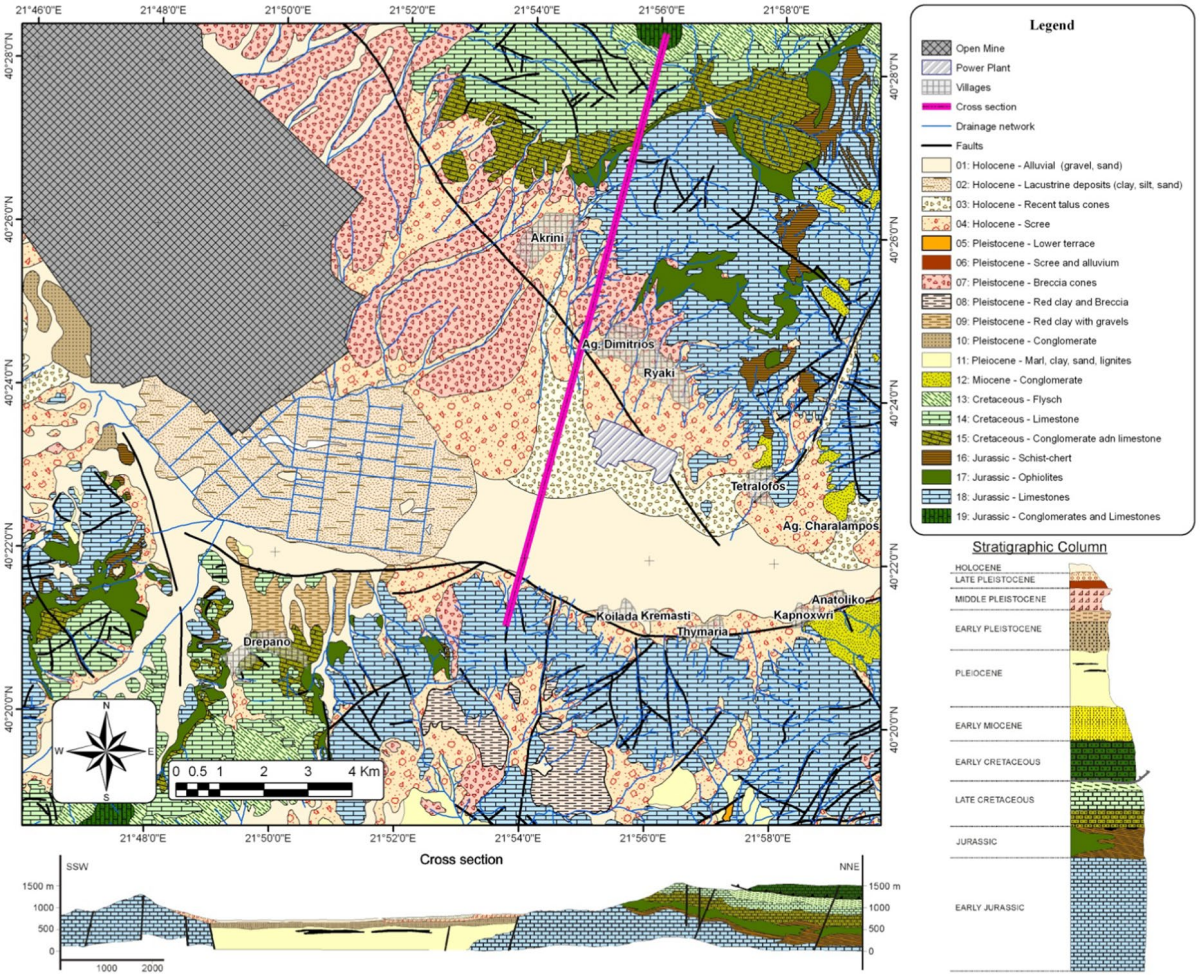
Geological map of the study area (Kazakis et al., 2017)

The Sarigkiol basin is located in northwest Greece, and is the southern part of the larger basin of Ptolemaida, near the city of Kozani (Kazakis et al., 2018). The Agios Dimitrios Power Plant is in the basin’s eastern section (Fig. 1). The lithological formations of the Sarigkiol basin belong to the Pelagonian geotectonic zone. The basement rocks are consisted of the Paleozoic metamorphic formations. The mountainous territory, around the perimeter of the basin, is composed of carbonate and ultramafic rocks, such as ophiolite complexes.

The wide area of the Sarigkiol basin consists of three main group formations. The first is the Triassic carbonate rocks with the overlaying ultramafic formations. The second concerns a sedimentary sequence. In specific, the lowlands of the basin are constituted by Neogene and Quaternary sediments. In detail, along the basin’s margins are detected the upper formations of the basin, consisting of conglomerates and gravels. At the inner parts of the basin, clay and sand formations are spotted. The final, third group, consisted of the Neogene marls, alternating with lignite deposits at the greater depths of the basin.

At the broader trench of Monastiri—Florina—Vegoritida—Ptolemaida to which the Sarigiol Basin belongs, two groups of faults prevail (Pavlidis, 1985). The first fault group presents a WNW-NNW direction and occurred during the Upper Miocene—Pliocene. It is responsible for the creation of the large depression of Florina-Ptolemaida-Kozani, with normal faults of NW–SE direction.

The second fault group presents a WNW-NNW direction and occurred during the Quaternary. It is responsible for the creation of smaller sub-basins and escarpments, perpendicular to the initial subsidence (Pavlides & Mountrakis, 1987). This category includes the escarpment of Komanos, which defines the Sarigiol sub-basin. A detailed depiction of the geological, tectonic, and lithological characteristics of the basin is presented in Fig. 1.

Fly ash samples were retrieved from the Agios Dimitrios Power Plant, which is supplied by the largest mine in Greece, the South Field Mine. Marl samples were collected from the South Field Mine located in Kozani, Greece.

### Sample preparations and leaching experiments

To carry out the leaching experiments, representative amounts of both fly ash and marl were separated and prepared for the mixtures. Five mixtures (F1M-F5Μ) were prepared using varied weight ratios (Table 1). F1M and F2M represent the initial materials, 100% fly ash (FA), and F2M 100% marl (M), respectively. The remaining mixtures-F3M (75FA-25 M), F4M (50FA-50 M), and F5M (25FA-75 M)-involve different concentrations of fly ash and marl, with a gradual evaluation in the marl percentage, under different pH conditions.

**Table 1:** Percentages of Fly ash-Marl mixtures (% w/w) and their initial pH

Sample	Fly ash	Marl	Initial pH
F1M	100	0	12,5
F2M	0	100	8,7
F3M	75	25	13,3
F4M	50	50	13,4
F5M	25	75	13,4

The samples were subjected to leaching based on the EN-12457/1-4 (2003) standard for leaching experiments, a widely recognized protocol for characterizing the leaching behavior of waste materials, suitable for granular solid waste, ensuring a systematic and standardized approach to data collection (Izquierdo & Querol, 2012).

The EN-12457/1-4 (2003) leaching test consisted of two consecutive leaching phases, the leaching stage, and the liquid and solid material separation stage, on room temperature (20 ± 5 °C). The solid material collected was placed in a bottle, an appropriate amount of distilled water was added for the leaching test. The liquid: solid ratio was adjusted according to the 10/kg (10:1) standard. The samples were placed for 24 ± 0.5 h in the rotary shaker of liquid–solid mixture, to settle on balance the liquid and solid phases. The flask remained undisturbed for 15 ± 5 min, to the suspended particles to settle. After liquid/solid centrifugal separation was applied at high speeds (> 4000 rpm) for about 5 min, the solution was filtered through a 0.45 μm membrane filter.

Following that, leachates were examined, and the pH levels of the initial samples were recorded (Table 1). The Standard Analytical Method 4500-H + B was used for the pH determination of the leachate. The initial fly ash and marl mixtures exhibited pH values ranging from 8.7 to 13.4. To achieve the target pH values (6, 8, 10, and 12 for the leaching evaluations), particular amounts (SV, ml) of standard sodium hydroxide (NaOH) and hydrochloric acid (HCl) solutions were used (Table 2).

**Table 2:** Adjustment of the target pH value for the leaching experiments

Sample/Mixture-Target pH	Fly Ash—Marl (% w/w)	HCl (ml)	NaOH (ml)
F1M_6_	100–0	37.2	
F2M_6_	0–100	21.0	
F3M_6_	75–25	33.5	
F4M_6_	50–50	34.3	
F5M_6_	25–75	18.8	
F1M_8_	100–0	36.0	
F2M_8_	0–100	28.8	
F3M_8_	75–25	33.0	
F4M_8_	50–50	27.4	
F5M_8_	25–75	24.5	
F2M_10_	100–0	37.4	
F3M_10_	0–100		3.3
F4M_10_	75–25	32.0	
F5M_10_	50–50	24.6	
F1M_10_	25–75	21.2	
F2M_12_	100–0	22.0	
F3M_12_	0–100		5.3
F4M_12_	75–25	18.3	
F5M_12_	50–50	28.5	
F1M_12_	25–75	35.7	

Cr (VI) has a high level of toxicity, hence measuring and monitoring its presence in real samples—like wastewater—is important. Consequently, following the execution of the leaching processes, the concentration of Cr (VI) was both in the initial fly ash and marl mixtures and the mixtures adapted to the specific pH conditions, as hazardous material of the leachates. Its concentrations were measured by spectrophotometry with diphenylcarbazide method using an atomic absorption spectrophotometer at 525 nm (APHA-AWWA-WEF, 1999; Hua et al., 2009). Diphenylcarbazide analysis is one way to examine Cr VI) in water samples (Papassiopi, et al., 2009). The assessment of Cr (VI) concentration in the solution involves its interaction with diphenylcarbazide (DPC) in an acidic environment, leading to the formation of a complex compound with a pink-violet color. The concentration of hexavalent chromium determines the color’s intensity. Subsequently, the quantification of this complex solution is conducted using a UV–VIS spectrometer at 540 nm. Laboratory analyses were conducted at the Department of Mineralogy-Petrology-Economic Geology, School of Geology, Aristotle University, Thessaloniki, Greece.

## Results–discussion

### Leaching experiments—Leachate chemical analyses

#### Leachate chemical analyses on initial samples

The pH of the solution is one of the most important variables affecting the leaching behavior of the elements (Adamidou et al., 2007; Komonweeraket et al., 2005). The amount of fly ash in the mixtures and the pH levels had a significant impact on the leachability of Cr (VI).

The pH of the initial fly ash sample F1M (100% FA) suspended in deionized water was 12.5, whereas the pH of the marl sample F2M (100% M) was 8.7. There have been previous reports (Georgakopoulos et al., 2002; Querol et al., 1995) of similar pH values in fly ash samples from the Agios Dimitrios Power Station. The increased concentrations of CaO and Ca(OH)_2_ (Moreno et al., 2005), as well as the hydrolysis of metal oxides like calcium, sodium, magnesium, and potassium that are formed during the combustion of coal (Izquierdo et al., 2011), are the main causes of the higher pH values seen in the leachates from the fly ash of Agios Dimitrios.

**Fig. 2:**
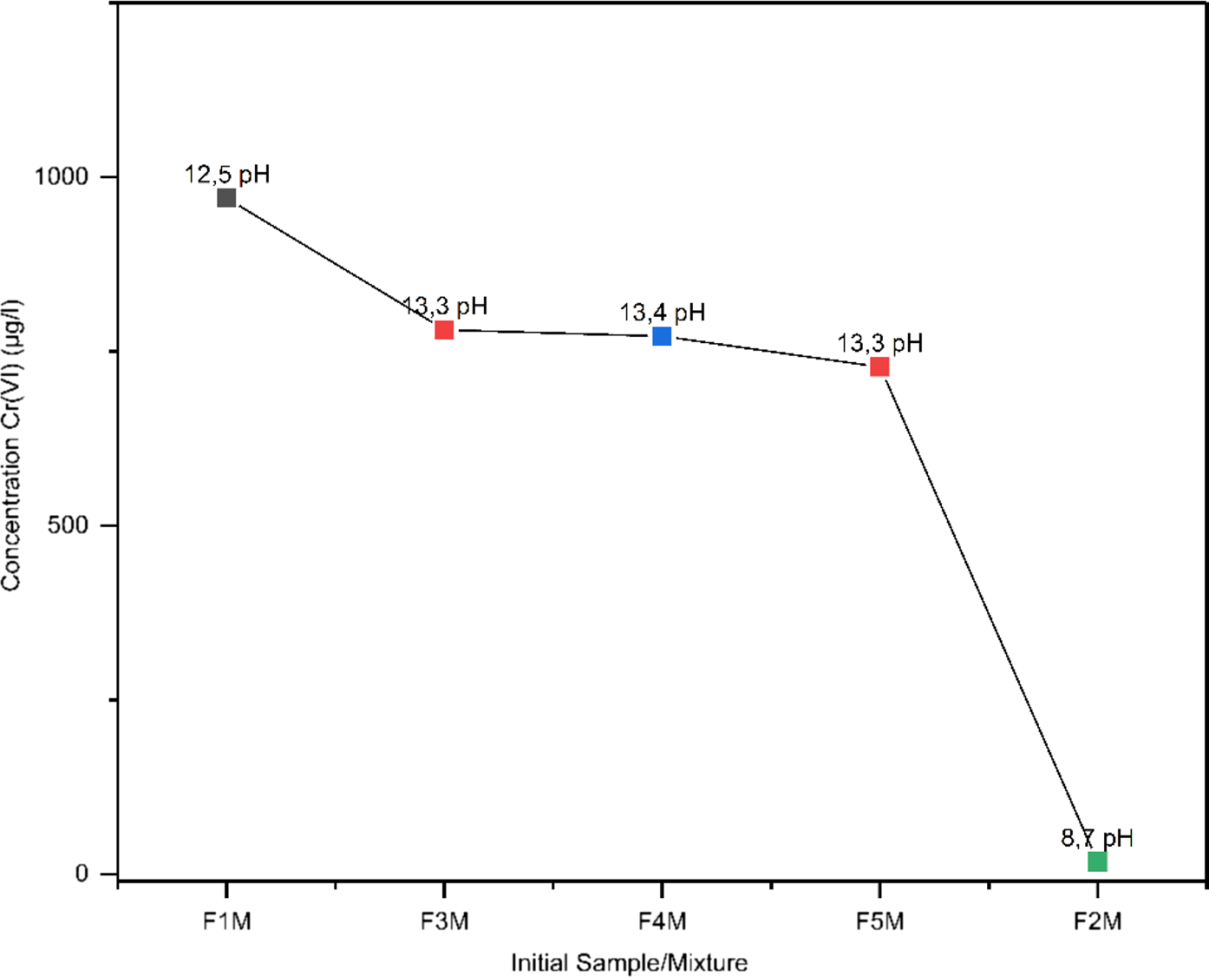
Variation at initial pH of Cr(VI) (μg/L) in initial mixtures of fly ash and marl

At first, the leachability of Cr(VI) was examined from the initial fly ash and marl samples, as well as from their mixtures (Table 3). In the initial fly ash (F1M12.5), higher leachability can be observed at high Cr(VI) concentrations (970 μg/L), while in the initial marl (F2M8.7), the leaching of Cr(VI) is significantly lower (18 μg/L). In all mixtures (F3M-F5M)13,3-13,4, a high concentration of Cr(VI) is observed, with the highest being in the mixture F3M13.3 (781 μg/L). A gradual decrease in leaching can be identified as the percentage of fly ash declines (F5M13.3) (728 μg/L) (Fig. 2). Leaching behavior showed sensitivity to the percentage of initial materials in the mixtures, with higher concentrations of fly ash showing a higher environmental footprint of Cr(VI).

**Table 3:** Hexavalent chromium in initial mixtures of fly ash and marl (μg/L)

Mixtures/Samples	Initial Cr(VI)
F1M	970
F2M	18
F3M	781
F4M	772
F5M	728

According to the lignite mineralogy of the study area, the higher fly ash Cr(VI) concentration can be partially explained either by the slow kinetics of diffusion of Cr(VI) from the bulk of fly ash particles, such as ettringite, to their surface, or by the slow oxidation of Cr(III) to Cr(VI). However, Cr (III) is comparatively resistant to oxidation and dissolved oxygen oxidation at ambient temperature is extremely sluggish (Apte et al., 2006; Van der Weijden & Reith, 1982). It is important to mention that the increase in the concentration of Cr(VI) can be affected by the release of additional components that interact with it or that its concentration is reduced to some extent by the presence of other elements. At neutral pH, the presence of sulfate ions or barium can affect the leaching, adsorption, and desorption processes of chromium (Fruchter et al., 1990), while the presence of iron or organic materials can be involved in the process of reducing hexavalent to trivalent chromium (Apte et al., 2006; Van der Weijden & Reith, 1982). Furthermore, the ability to leach Cr under neutral pH conditions has been noted to result from the dissolution of mineral phases in which Cr is bound, specifically Cr_2_O_3_(s) (Komonweeraket et al., 2011). In both acidic and alkaline conditions, there is a different variation in Cr concentrations that can be attributed to the dissolution of these oxide/hydroxide minerals.

**Fig. 3:**
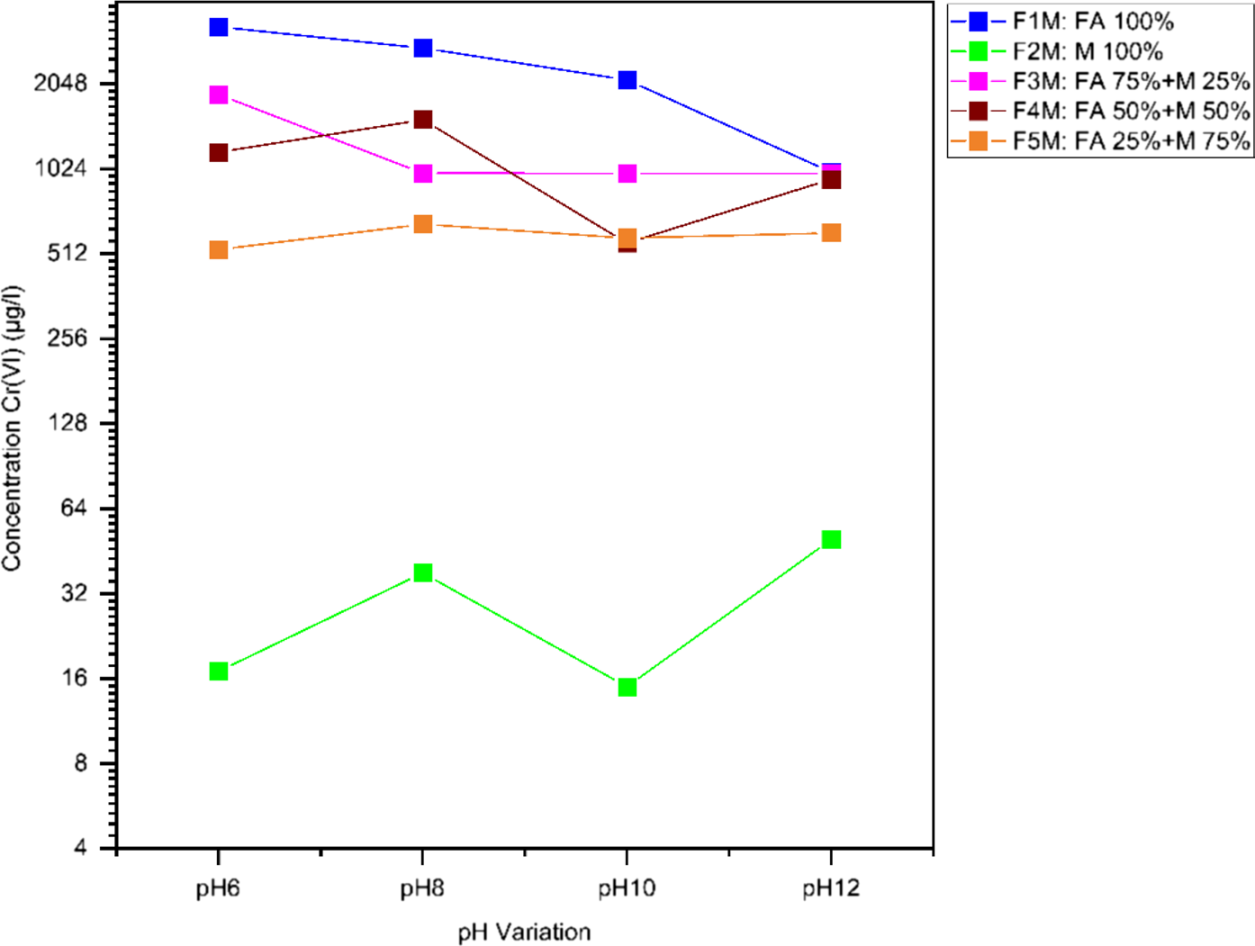
Variation at pH 6–12 of Cr(VI) (μg/L) in the studied mixtures

**Fig. 4:**
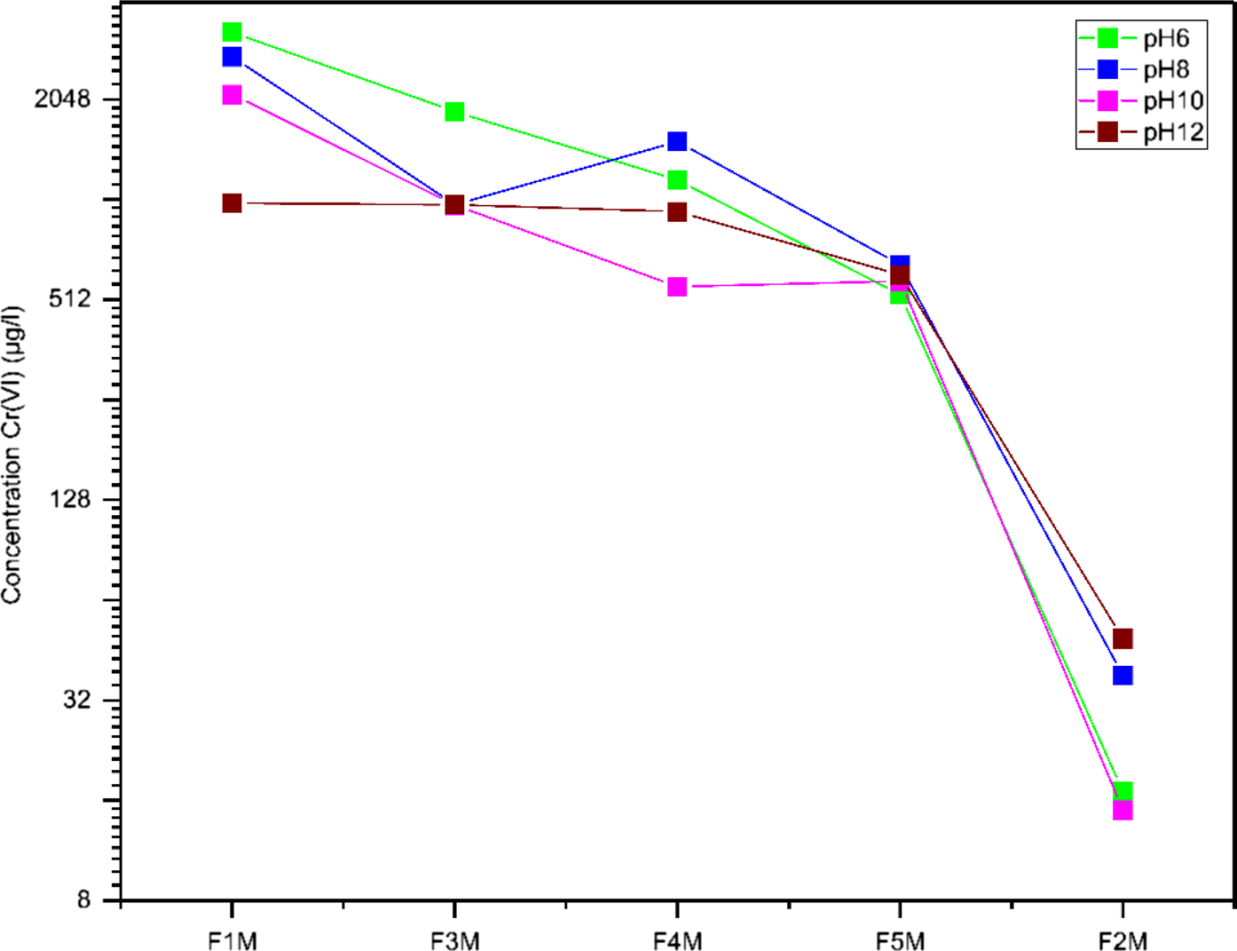
Variation of fly ash/marl concentration on leaching Cr(VI) (μg/L) under specified pH conditions

The results of the leaching experiments were analyzed in two phases. In the first phase, constant proportions of the studied mixtures were maintained while varying the pH conditions to the target values of 6, 8, 10, and 12. In the second phase, the pH conditions were maintained while varying the proportions of the studied mixtures, with a gradual increase of fly ash in the mixtures (Figs. 3 and 4).

#### Effect of pH on leaching Cr(VI) process

In the fly ash (FM1), leaching of higher concentrations of Cr (VI) can be observed, with the highest concentration being at pH 6 (38,711 μg/L). Cr (VI) shows a high concentration at both pH 8 and 10, with the concentration being observed at 2751 μg/L and 2121 μg/L respectively, followed by a decrease in leaching at pH 12, although its concentration remains high (998 μg/L). In the marl sample (FM2), Cr(VI) concentrations were significantly low, with the highest concentration being observed at pH 8 (38 μg/L), and at pH 12 was found at concentration below the detection limit (Table 4) (Fig. 3).

**Table 4:** Chemical analysis of mixtures/samples leaching fluids at pH 6–12 (μg/L)

Sample/Mixture	Detection limit	pH 6	pH 8	pH 10	pH 12
F1M	100	3264	2751	2121	998
F3M		1881	989	987	988
F4M		1173	1530	560	942
F5M		530	652	583	608
F2M		17	38	15	100

A critical pH range of 6 to 8 was detected due to the release of Cr (VI). In all mixtures, above the mentioned critical levels, the release of Cr (VI) decreased progressively, while at more acidic pH conditions, the release of Cr (VI) was significantly increased. (Fig. 3) The leachate of the F3M mixture shows high Cr (VI) concentrations throughout the pH 6–12 range, with the highest value observed at pH 6 (1881 μg/L), followed by a gradual decrease in leaching up to pH 10 and pH 12 (987 and 988 μg/L). In the leachates of F4M mixture it continues to present high concentrations of Cr (VI) at pH 6 and pH 8 (1173 μg/L and 1530 μg/L respectively), while as the pH inclines, the concentration of Cr (VI) in the leachate sample decline at pH 10 (560 μg/L) and incline at pH 12 (942 μg/L). In the leachate of the F5M mixture, lower concentrations of Cr (VI) are observed, with a higher concentration at pH 8 and pH 12 (652 and 608 μg/L) (Table 4).

#### Effect of fly ash/marl on leaching Cr (VI) under specified pH conditions

At pH 12, both fly ash and mixtures with a high percentage of fly ash have significant concentrations of Cr (VI) in leachates F1M-F5M (Table 4) (Fig. 4). The maximum concentration of Cr (VI) in leachates was observed in the F1M sample with a value of 998 μg/L, which gradually decline as the fly ash content in the mixtures decreases, reaching 608 μg/L n the mixture F4M with 25% w/w fly ash. The marl sample (F2M) shows the concentration of Cr(VI) below the detection limit leaching at pH value 12. It is clear from the previous statements that at pH value 12, the fly ash is responsible for the leaching of Cr (VI).

At pH 10, Cr (VI) was withdrawn at the liquid phase from the fly ash (F1M) sample at a significant high concentration (2121 ppm) and was still identified at great concentrations in the leachate of fly ash-marl mixtures F3M (987 ppm), F4M (942 μg/L), and F5M (560 ppm), clearly indicating fly ash as soluble Cr source. It is worth stating that in regard to the marl (F2M) sample Cr leaches (VI) at significantly lower concentrations at pH10. In contrast, at pH 8, Cr (VI) shows a mixed behavior influenced by both fly ash and marl content in the mixtures. Cr (VI) extracted in the liquid phase from fly ash (F1M) at pH 8, at an extremely high concentration (2751 ppm), and it is still present in significant concentrations in the leachate of fly ash-marl mixtures F3M (989 ppm), F4M (1530 ppm) and F5M (652 ppm), clearly indicating both fly ash and marl as soluble Cr source. It is worth mentioning that only a small amount of Cr (VI) was leached for marl sample F2M (38 ppm). At pH 6, the leaching trend is equivalent with pH 10 and pH 12, with a gradual decrease in Cr (VI) concentration as the percentage of fly ash in the mixture decreases, also indicating fly ash as its source. The maximum concentration of Cr (VI) in leachates was observed in the F1M sample with a value of 3264 ppm, which gradually decrease as the fly ash content in the mixtures decrease, reaching 530 in the mixture F5M with 25% w/w fly ash. The marl sample (F2M) shows low Cr (VI) leaching at pH value 6 (17 ppm) (Fig. 4).

It is clear from the analysis of the effect of fly ash to marl ratio at specific pH conditions that fly ash was the main cause of Cr (VI) leaching in the pH range of 6 to 12, demonstrating fly ash as a soluble source of Cr. The leaching profile for coal fly ash found in another study was different from this leaching pattern (Zandi & Russell, 2007). In this study, they reported the pH-dependent leaching of Cr_total_ (Cr (III) and Cr (VI). An acidic pH condition has been found to release chromium, but only Cr (III) was reported to be released in this environment. In fact, the release of reducing agents such as iron could be the main factor leading to lower concentrations of Cr (VI) in leachates at acidic pH conditions. In contrast, under alkaline conditions where only chromates dissolve in water, the study noted at pH 10, the highest leaching of Cr. The variation of these leaching patterns with increased alkalinity may be related to the different type of lignite.

### Potential limitations of case study

The research used the leaching standard EN-12457/1-4 (2003), providing a standardized and repeatable approach to assess the environmental impact of fly ash, marl, and their mixtures due to the high leachability of Cr(VI). However, it is important to consider several limitations when interpreting the results.

The study was conducted under controlled laboratory conditions, which may not fully represent real environmental conditions. While the study examines different pH conditions, the selected pH ranges may not fully cover the extreme conditions that may exist in some environmental settings. Factors such as temperature fluctuations, microbial activity, and other physical processes could affect leaching behavior differently in the situ. Furthermore, it is important to emphasize the short duration of the study, with other factors such as weather and changing environmental conditions not accounting for long-term trends or variations in fly ash and marl leachability characteristics. The leaching experiment suggested that the release of Cr(VI) from the mixtures and their solubility were strongly affected by changing the percentage of fly ash in the mixture. The diversity in the leachability profile can be attributed to the different fuel origins, but also to the different composition of the fly ash and marl depending on their source.

Various approaches can be used to detect heavy metal contaminants, such as Cr (VI), which can be analyzed in situ. The diphenylcarbazide assay, used to measure Cr (VI) concentrations, is one of the most common methods used to determine Cr (VI) in water samples (Andrade et al., 1996; de Andrade et al., 1983). However, although some measurement methods, including the diphenylcarbazide assay of hexavalent chromium, are simple, it is important to mention that there may be an overestimation or underestimation of the concentration of the element (Georgaki et al., 2023; Onchoke & Sasu, 2016). Alternatively, there are many more expensive, time-consuming, and technically complex procedures available that are used to determine Cr, including, ion chromatography (IC), atomic absorption spectrometry (AAS), inductively coupled plasma optical emission spectrometry (ICP-OES), inductively coupled plasma spectrometry plasma-mass spectrometry (ICP-MS).

Finally, the study can determine the degree of leaching but does not provide a comprehensive risk assessment. More extensive analysis of hexavalent chromium leachability is needed, addressing the limitations, with the applicability of findings to different environmental contexts, using advanced methodologies.

## Conclusion

The study aimed to examine the leaching behavior of Cr (VI) from fly ash, marl as well as a combination of the two. A broad pH range between 6 and 10 was observed, with acidic pH values enhancing the leaching of Cr(VI).On the other hand, at pH conditions of 10 and 12, a gradual decrease in the leachability of the element was observed. The environmental footprint of chromium in the study area is highlighted, particularly in fly ash samples and mixtures containing higher concentrations of fly ash. In both the marl samples and a mixture sample, no significant concentration of Cr (VI) is observed. As a result, improper management of fly ash contributes to the environmental impact of the Sarigiol basin.

The method of data extraction and result evaluation applied in this work can be a tool for future calculations and evaluations of samples with high percentages of Cr (VI) content in corresponding geological/lithological/hydrogeological regimes. The method can be used as a guide to prevent Cr (VI) contamination of groundwater and help design appropriate practices to remediate any contaminated aquifer. Furthermore, the methodology can be applied globally in similar environmental cases to distinguish the source of origin of Cr (VI) (geogenic origin or anthropogenic intervention), especially near industrial activity. However, our future conducted research, will include additional analysis of Cr(VI)-affected groundwater samples and human urine samples, which will highlight and further elucidate the specific processes and release mechanisms involving the presence of Cr(VI) in groundwater and how they may affect human health.
